# Aggregatibacter actinomycetemcomitans infection in a 15-year-old boy with pulmonary empyema: a case report and review of literature

**DOI:** 10.1186/s13052-023-01429-4

**Published:** 2023-03-31

**Authors:** Maria Alessia Mesturino, Carol Bitetti, Anna Clemente, Andrzej Krzysztofiak, Laura Lancella, Roberta Lombardi, Laura Cursi, Elena Boccuzzi, Anna Maria Musolino, Alberto Villani

**Affiliations:** 1 Unit of Emergency Pediatrics, Department of Emergency, Admission and General Pediatrics, Bambino Gesù Children’s Hospital (IRCCS), Rome, Italy; 2 University Hospital Pediatric Department, Bambino Gesù Children’s Hospital (IRCCS), Rome, Italy; 3 Department of Maternal, Infantile, and Urological Sciences, Faculty of Medicine and Dentistry, Sapienza University of Rome, Rome, Italy; 4 Infectious Diseases and Immunoinfectivology Unit, University Hospital Pediatric Department, Bambino Gesù Children’s Hospital (IRCCS), Rome, Italy; 5 Unit of Emergency Radiology, Department of Diagnostic Imaging, Bambino Gesù Children’s Hospital (IRCCS), Rome, Italy; 6 Unit of General Pediatrics, Department of Emergency, Admission and General Pediatrics, Bambino Gesù Children’s Hospital (IRCCS), Rome, Italy

**Keywords:** Aggregatibacter actinomycetemcomitans, HACEK, Pulmonary empyema, Chest wall abscess, Drainage fluid culture, Antimicrobial therapy, Case report

## Abstract

**Background:**

Aggregatibacter actinomycetemcomitans (Aa), previously known as Actinobacillus actinomycetemcomitans, is a slow-growing Gram-negative coccobacillus, member of the HACEK group of bacteria colonizing oral flora. Besides causing infectious diseases in the oral cavity such as dental caries and periodontitis, it is responsible for severe extra-oral infections secondary to hematogenous spread or aspiration, such as endocarditis, soft tissue abscesses and osteomyelitis. The diagnosis depends on prolonged bacterial culture of biological material obtained through biopsy. Aa is susceptible to most antibiotics but complete eradication often requires a long term treatment.

**Case presentation:**

We report the case of a 15-year-old previously healthy boy diagnosed with both pulmonary empyema and subphrenic chest wall abscess caused by Aa. He was admitted to our Pediatric Emergency department for evaluation of a right mass associated with marked asthenia and dry cough. After radiological findings etiological diagnosis was made by culture of fluid drainage of pleural empyema. He started empirical antibiotic therapy with intravenous piperacillin/tazobactam, whose sensibility was confirmed by the antibiogram, then, for occurrance of hepatopathy it was switched to ciprofloxacin: the patient almost completely recovered after 6-month therapy.

**Conclusions:**

Extra-oral infections caused by Aa are extremely rare, especially in children, and not well described yet. To our knowledge, there is only another similar case described in literature. However, the case described in our manuscript represents the only one presenting with pulmonary empyema without involvement of lung parenchyma in children. We also conducted a brief review of published cases of Aa infection in the pediatric population. This case report reminds us the importance of an accurate inspection of the oral cavity during the examination of pediatric patients.

## Background

Aggregatibacter actinomycetemcomitans (Aa) is a slow-growing, capnophilic, Gram- negative coccobacillus, representing an oral commensal in over 30% of apparently healthy children. It was first described in 1912 by Klinger, known as Actinobacillus actinomycetemcomitans, and in 2006 it received its current name [1].

It is a member of the HACEK group of bacteria (Haemophilus including paraphrophilus and aphrophilus species, Aggregatibacter, Cardiobacterium hominis, Eikenella corrodens and Kingella kingae), that colonize the oral-pharyngeal flora [2].

They are associated with infectious diseases in the oral cavity such as dental caries and periodontitis, by an arsenal of virulence factors, such us the ability to form biofilms [3]. Aa is also able to produce toxins, like the cytolethal distending toxin (CDT) and leukotoxin [4]. The highly leukotoxic clone, which is named the JP2 clone and which belongs to the group of serotype b strains, is strongly associated with rapidly progressing forms of severe periodontitis [5].

Hematogenous spread from infected tissues of the oral cavity can affect heart valves and soft tissues including brain and lung, joints and distal bones.

HACEK organisms account for 6% of all adult and pediatric cases of bacterial infective endocarditis [6].

There are several risk factors associated with systemic diffusion, such as malnutrition, poor oral hygiene, injury or inflammation of oral cavity, DM (diabetes mellitus), immunosuppression (chronic granulomatous disease), aspiration, local tissue damage (neoplasia, trauma, irradiation).

Multiple different clinical manifestations have been described, associated to various anatomical sites. It frequently mimics malignancy, tuberculosis, or nocardiosis: it spreads continuously and progressively.

It is not a prompt diagnosis. Imaging methods such as computed tomography (CT) scan, or magnetic resonance (MR) can be used. The cornerstone of diagnostic procedure involves the collection of biological material to perform prolonged bacterial cultures in anaerobic conditions (incubation of 72–96 h).

Complete eradication of Aa often requires a long-term antibiotic treatment. Surgery and tissue sampling are necessary for a definitive diagnosis in complicated cases.

Most infections are polymicrobial, involving other aerobic and anaerobic bacteria. Co-isolates depend on the source or site of infection and include Actinomyces, aerobic and anaerobic Streptococci, Staphylococcus, and Enterobacteriaceae [7].

We report a case of Aa invasive infection in a 15-year-old previously healthy boy, who was admitted to Bambino Gesù Children’s Hospital of Rome. It represents the only case reported in current literature of a pediatric patient presenting with pulmonary empyema and subphrenic abscess caused by Aa infection, without involvement of pulmonary parenchyma.

## Case presentation

A 15-year-old boy from the Philippines, with a low socio-economic level and no significant past medical history, presented to our Pediatric Emergency Department for evaluation of a right abdominal mass appeared during the last 7 days, associated with occasional dry cough and marked asthenia. No fever, weight loss or recent history of trauma were reported.

On physical examination he was comfortable, except for mild respiratory distress. Respiratory rate was 20 breaths/minute, heart rate 100 beats/minute, blood pressure 107/74 mmHg, temperature 36.8 C and oxygen saturation was 97% in room air. His weight was 40,5 kg.

On the examination of the oropharynx, two caries were noted affecting inferior molars. Small latero-cervical lymph nodes were palpable bilaterally. Chest auscultation revealed decreased breath sounds in the mid-apical right lung and some homolateral basal crackles. Cardiac objectivity was normal.

Abdomen physical examination showed, in correspondence of the right hypochondrium, a roundish bulging of taut-elastic consistency, with a diameter of 8 cm, painful at palpation, with slightly warm overlying skin.

A point-of-care lung ultrasound showed a sub-pleural anechoic lesion in the mid- apical right chest area, with some hyperechoic spots in the context, and a small amount of right pleural effusion.

**Fig. 1:**
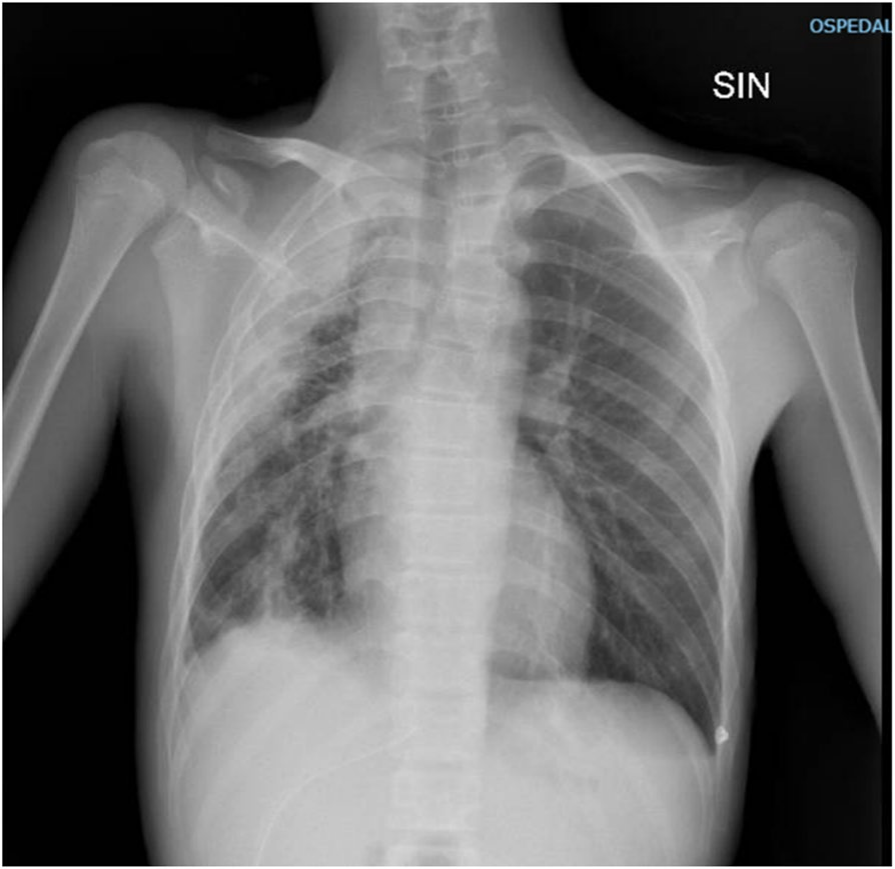
Chest-X-Ray. Antero-posterior Chest-X-Ray showing a semi-moon radiodensity in the right upper and middle lung field and disventilative/dystelectasic phenomena of the adjacent lung, together with a slight homolateral shift of mediastinum

Chest-X-ray revealed a semi-moon radiodensity in the upper and middle right lung field and atelectasis of the adjacent lung, together with a slight homolateral shift of mediastinum (Fig. 1).

**Fig. 2:**
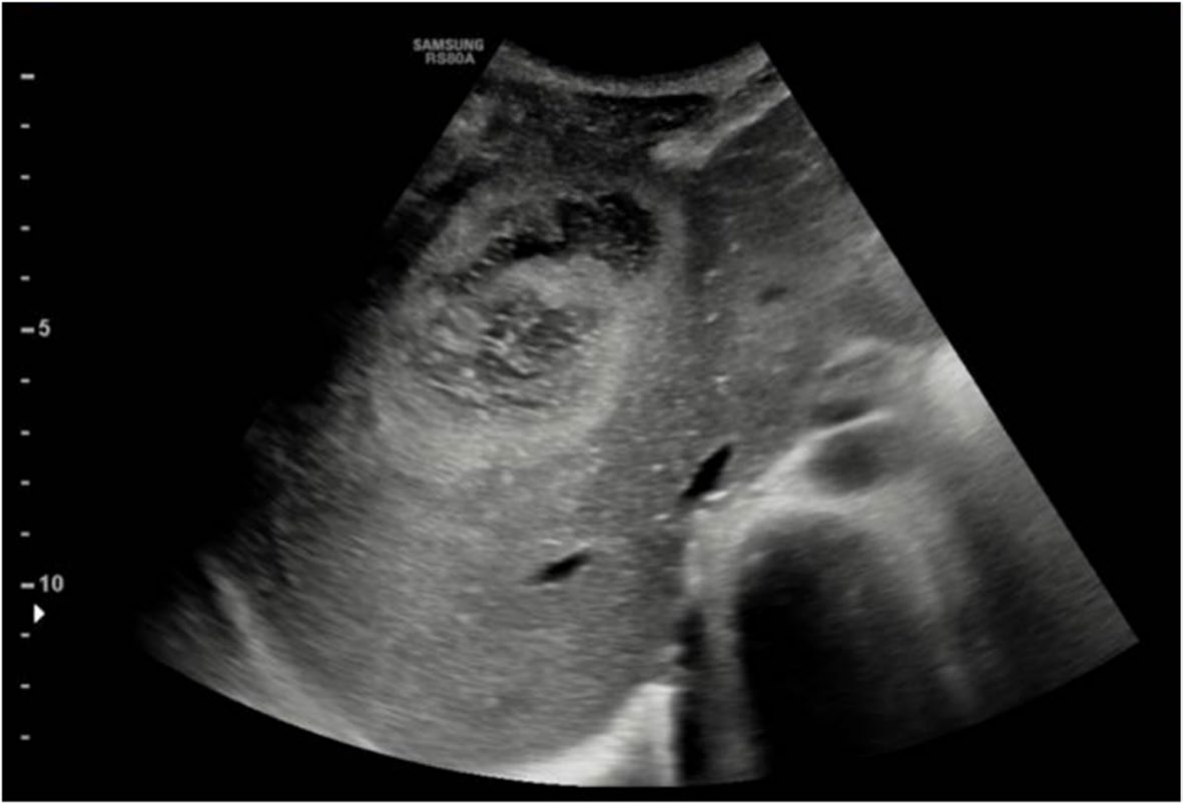
Abdominal ultrasound. An abdominal ultrasound scan showing a 9 × 6 cm bilobed mass, with fluid-corpuscolated content, apparentlyextra-hepatic and compressing the adjacent hepatic parenchyma

Abdominal ultrasound showed a 9 × 6 cm bi-lobed mass, with fluid-corpuscolated content, delimited by a color-doppler hyper vascularized wall. Such a lesion, of about 9 × 6 cm, seemed apparently extra-hepatic, though compressing the adjacent hepatic parenchyma (Fig. 2).

Complete blood count demonstrated a white blood cells count of 16,97 × 10^3 cells/µL (78% neutrophils), with normal haemoglobin and platelets. Blood chemistry revealed C-reactive protein 3.56 mg/dL, lactic dehydrogenase 370 U/L, gamma glutamyl transpeptidase 83 U/L, with normal electrolytes and hepatorenal function indices.

The patient was admitted to the Infectious Diseases Unit of our Institution for further evaluation, with the clinical suspicion of infectious disease versus malignancy.

**Fig. 3:**
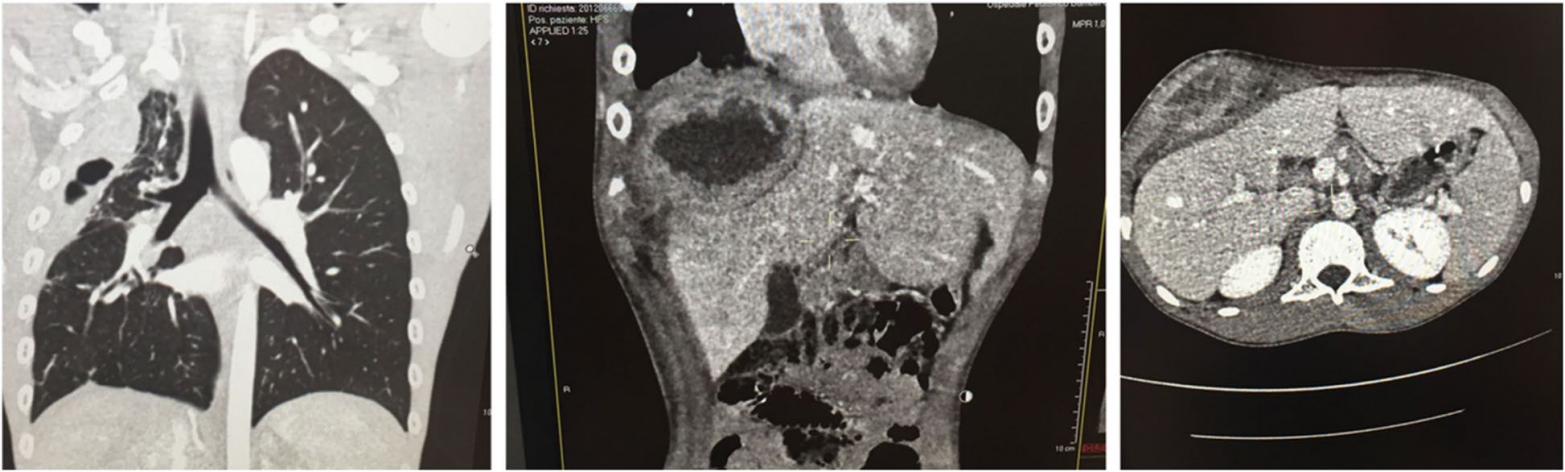
Thoracic CT scan. On the left a thoracic CT scan showing a right extrapulmonary lesion consisting of necrotic tissue with air- filled bubbles inside it. The abdominal scans, on the right of the figure, reveal an extra- hepatic mass-like lesion of 11.8 × 6 × 13 cm with central colliquation dislocating the II, IV and VII segment of the liver. CT, computed tomography

A thoraco-abdominal computed tomography with contrast showed, in correspondence to the right chest wall, an extra-pulmonary lesion consisting of necrotic tissue with air-filled bubbles inside it, extended from the apical pleura to the sixth intercostal space and a right hilar lymphadenopathy of 2 × 1.4 cm without signs of colliquation. The abdominal scans revealed, in the site of the clinically appreciable swelling, an extra-hepatic mass-like lesion of 11.8 × 6 × 13 cm with central colliquation and peripheral ring enhancement dislocating the II, IV and VII segment of the liver (Fig. 3). Such findings were confirmed and better characterized at MR.

Rhino-pharyngeal swabs for respiratory viruses (including SARS-Coronavirus 2), Mycoplasma and Chlamydia pneumoniae were negative. Quantiferon test and viral serologies revealed negative too. Blood, sputum, urine and stool cultures didn’t show growth of any microorganism. Galactomannan antigen detection and serologic tests for Entamoeba histolytica and Echinococcus gave no remarkable results.

The following tumour markers were tested: chromogranin A, carcinoembryonic antigen, human alfa chorionic gonadotropin, alpha fetoprotein, urine metanephrines, vanillylmandelic and homovanillic acid. They were all negative.

The 1st level immunological tests (immunoglobulins classes, lymphocyte subpopulations, Nitroblue Tetrazolium Test, Dihydrorhodamine Test) resulted in the range of normality.

The patient underwent a biopsy of the abdominal lesion and positioning of an aspiration drainage. The pathological finding from the biopsy was inflammatory granulation tissue without the presence of malignant cells but with the presence of sulphur granules, which raised the suspicion for actinomycosis. Three days later Aa was isolated from the culture of the drainage fluid. This microorganism was susceptible to most classes of antibiotics such as beta-lactames, quinolones and sulphonamides.

**Fig. 4:**
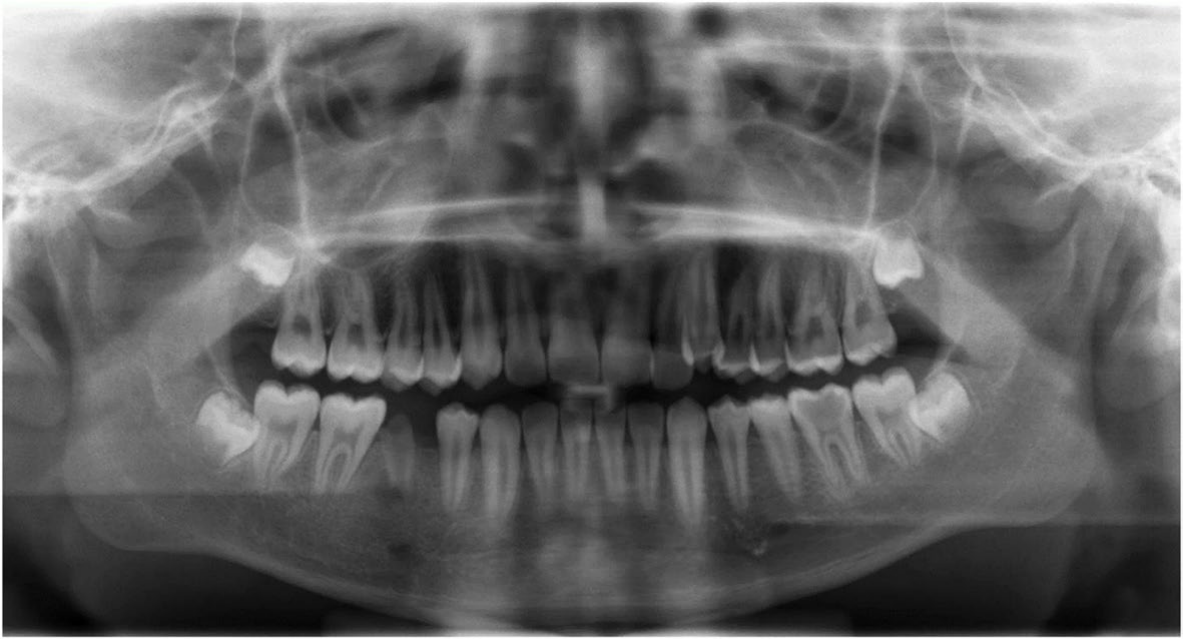
Panoramic X-ray. A panoramic X-ray showing partial destruction of the 45 with resorption of the root apex and hypodensity of the crown of 36 with no evidence of periapical alterations

Our patient underwent a panoramic X-ray (Fig. 4) and a dental consultation. The panoramic X-ray showed partial destruction of the 45 with resorption of the root apex and hypodensity of the crown of 36 with no evidence of periapical alterations. At a careful periodontal examination, no signs of periodontitis were detected. The appropriate conservative dental treatment will be carried out at the end of the antibiotic therapy.

In order to exclude concomitant endocarditis, a complete echocardiogram which showed no evidence of vegetation.

The patient started empirical antibiotic therapy with intravenous piperacillin/tazobactam, whose sensibility was confirmed by the antibiogram. After one month of therapy, it was switched to intravenous ciprofloxacin for occurrence of hepatopathy.

After two weeks our patient was discharged in good clinical condition and continued oral ciprofloxacin for a total duration of six months.

Follow-up Chest-X-Ray and abdominal US performed three and six months later respectively, showed near complete resolution of both thoracic and abdominal lesions, though remaining a mild restrictive pneumopathy.

### Timeline

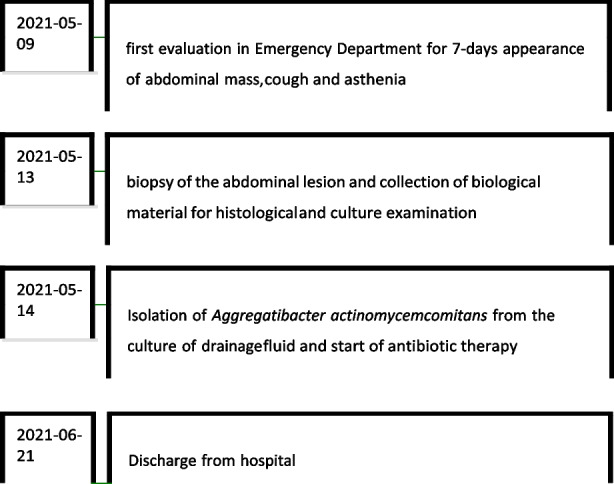



## Discussion and conclusion

Aa is a slow-growing capnophilic Gram-negative coccobacillus which was first isolated by Klinger in 1912, member of HACEK group of gram-negative bacteria [2]. Its name derived from the frequent association with Actinomyces israleii in actinomycosis. It is part of the normal human oral flora. It is associated with periodontal infection, dental disease, as well as for systemic infections, endocarditis, soft tissue abscesses; brain abscesses, osteomyelitis and pneumonia are less common [7].

We conducted a Medline-type bibliographic search to identify documented cases of pediatric infections caused by Aa, excluding those involving only the oral cavity (dental caries, periodontal disease) and including systemic infections, remote infections and those with local regional involvement (even in the facial district). Key search terms were ‘Aggregatibacter actinomycetemcomitans infection’ or ‘Case report of Aggregatibacter actinomycetemcomitans associated disease’, using filter for age (Child: birth-18 years) (Table 1).

**Table 1:** Review of cases of systemic infections caused by Aggregatibacter actinomycetemcomitans in pediatric population

	**Case 1 **^**8**^	**Case 2 **^**8**^	**Case 3 **^**8**^	**Case 4 **^**9**^	**Case 5 **^**10**^	**Case 6 **^**11**^	**Case 7 **^**12**^	**Case 8 **^**13**^	**Case 9 **^**14**^	**Case 10 **^**14**^
age (years) /sex	10/F	17/M	13/M	12/M	8/F	14/F	9/F	12/M	10/M	16/F
underlying disease	none	CP	none	none	residual tracheal–cutaneous fistula	none	none	none	none	none
periodontal disease or dental caries	no	yes	yes	no	yes	yes	yes	no	no	yes
duration of symptom	1 year	10 days	1 year	2 months	10 days	1 month and 2 weeks	1 month	2 weeks	1 year	7 months
symptoms/sign	no	fever	fever	shoulder pain	bloodtinged sputum, reduction of SaO2	cough with yellowish sputum, dyspnea, fever	Bulging chest wallmass	pain, swelling and purulent discharge from right great toe	Chest wall mass	upper back pain, fever, night sweats, weight loss
WBC (/µL)	8.300	20.000	12.500	10.900	11.500	18.000	8.400	6.200	8.300	not reported
clinical manifestation	chest wall mass, pneumonia	Pneumonia and empyema	pneumonia	Lung destructive lesion (mimick cancer)—bone destruction and soft tissue inflammation	Pleural pneumonia (empyema necessitatis)	Pleuralpneumonia extending to the chest wall and subphrenic area	Pneumonia/empyema	Osteomyelitis	Pneumonia, chest wall mass	Mediastinal and pulmonary abscesses, osteomyelitis of spine
sources of culture/ type of pathogen	chest wall biopsy	empyema	open lung biopsy	transthoracic tissue biopsy	Chest wall biopsy / AA and Actinomyces israeli	thoracoscopy	Chest wall biopsy	purulent material from the infected proximal end of thephalanx	Chest wall biopsy	thoracotomy/ AA and Actinomyces israelii, B. corrodens
duration of treatment	1 year	6 days	3 weeks	5 Months	1 year	3 months	1 year	13 weeks	1 year	1 month and 3 weeks
outcome	survived	died	survived	survived	survived	survived	survived	survived	survived	survived

	**Case 11 **^**14**^	**Case 12 **^**14**^	**Case 13 **^**15**^	**Case 14 **^**16**^	**Case 15 **^**17**^	**Case 16 **^**17**^	**Case 17 **^**18**^	**Case 18 19**	**Case 19 **^**20**^	**Case 19**^**21**^
age (years) /sex	17/F	15/F	9/M	11/M	11/M	14/M	4/F	1.5/F	16/F	18/F
underlying disease	CP	meningioma (neurosurgery removed)	none	none (autism)	DM1	NF1	none	Open valvuloplasty 6 months prior	none	none
periodontal disease or dental caries	yes	not reported	yes	yes	No	no	Yes	no	yes	yes
duration of symptom	10 days	not reported	1 month and 2 weeks	4 days	2 months	4 months	1 week	1 week	1 month	not reported
symptoms/sign	Cough, fever, dyspnea		cough, weight loss, and malaise	Intermittent fever, cough, right chest and rib pain	Intermittent left chest pain, left chest wall mass	fever, dry cough, anorexia, night sweats and a weight loss	fever, limp, toothache	fever	left facial mass	headeache, the confusion
WBC (/µL)	20.000	not reported	14.700	16.000	normal	14.100	12.000	normal	not reported	28.000
clinical manifestation	Empyema, left pleural effusion	Brain abscess	Pleuralpneumonia;bony destruction of one rib	right chest wall mass, pleuralpneumonia with periostitis of the interposed ribs	chest wall mass and pneumonia, with periosteal reaction of the left third rib	Pleuralpneumonia, chest wall mass, extending to the diaphragm. Lytic lesions involving interposed ribs with pathologic fractures	knee joint septic arthritis	Pulmonic valve vegetation	mass over the left mandibular area	sinogenic empyema,cerebritis, and subduralempyema
sources of culture/ type of pathogen	thoracentesis	fine needle aspiration /AA, Eikenella corrodens and H. aphrophiIus	open biopsy of the swelling/AA and A.israelii	Open biopsy	Lung biopsy	lung biopsy	knee joint aspiration	blood culture	Incision and drainage of the mass/ AA and Capnocytophaga spp	drainage
duration of treatment	1 week	1 month	4 months	6 months	1 y	> 3 months	5 weeks	4 weeks	not reported	40 days
outcome	died	survived	survived	Survived	survived	survived	survived	survived	survived	survived

Legend: *F* female *DM1* diabetes mellitus type 1, *CP* cerebral palsy, *Aa* Aggregatibacter actinomycetemcomitans

The main limitations of our case report are that current literature is poor of pediatric cases of infections by Aa and the manuscripts are often old. The strength of our manuscript is related to the uniqueness of the clinical presentation and the localization of infection in a pediatric patient, treated in a III level pediatric hospital.

Until January 2021, 20 cases of Aa infection were reported in the literature (including in the search patients aged 0 to 18), 11 females and 8 males, the age of the patients ranged from 1.5 to 18 years: in some cases it was the only isolated pathogen (15/20 cases), in 5 cases it was associated with other bacteria including Actinomyces israelii, Bacteroides ureolyticus (B. corrodens), Eikenella corrodens and Haemophilus. aphrophiIus, capnocytophaga species [8–21].

Aa infections may have different locations in children. There were described different pediatric cases with pulmonary involvement: 1 patients with pneumonia [8], 1 with empyema [14], 2 patients with pneumonia associated with chest wall mass [8, 14], 6 cases of lung consolidation with chest wall mass and a bony involvement (especially lytic lesions of the ribs), associated in 3 cases with a pleural effusion and in 1 case with a mediastinal and pulmonary abscesses [9, 14–17], 3 cases of pneumonia associated with empyema [8, 10, 12].

Our case is different from the others, the most similar is a case of a girl of 14 years old with lung consolidation, pleural infection extending to the chest wall and subphrenic area, but in our case there’s not concurrent pneumonia [8]. There are two cases described with brain involvement [14, 21]: one patient with a brain abscess after surgery, one case with subdural empyema.

Besides, one case of osteomyelitis [13], one with septic arthritis [18], one with endocarditis after valvuloplasty [19] and a one case of a soft tissue mass over mandibular area [20] are reported.

In our case, the Aa infection involved the visceral and parietal pleura, causing empyema, extending to the chest wall and subphrenic area.

All patients are immunocompetent, no specific immunological deficit were associated with infections, but in some cases there are underlying conditions associated with a major risk of Aa infections according to the related pathogenic mechanism, for example pulmonary infection by Aa is described in patients with an increased risk of aspiration: two cases of cerebral palsy [8, 14], one with residual tracheal–cutaneous fistula and tracheostomy with pulmonary infection by Aa [10]. In two patients Aa infection has been reported after heart and brain surgery [14, 19]. In one case Aa infection occurred in a patient with diabetes mellitus [14].

Clinical manifestations depends from localization, typically, the course of the infection is subacute, with fever, anorexia, weight loss, and night sweats, malaise, or bulging mass and local pain. In our case patient presented with a right abdominal mass with occasional dry cough and marked asthenia, without fever, weight loss or recent history of trauma were reported.

As the presentation is nonspecific for an infectious process, a differential diagnosis may be necessary, especially with malignancy, Mycobacterium tuberculosis infection and trauma [9].

Duration of symptoms ranged from 4 to 365 days, in this case they lasted 7 days. The precise pathogenesis of Aa infections is unknown. Because the Aa is a mouth commensal, presumably its invasive extension occurs via the mucous membrane resulting in haematogenous dissemination to the skin, joints, bones and disc spaces (due to enzymes in saliva that may modify mucosal surfaces to promote adhesion and colonization), or as a result of aspiration during dental procedure [22, 23].

In our case, we found that the Aa infection may have spread perpendicular to the chest wall, invading and breaking through the visceral and parietal pleura, extending to the chest wall and subphrenic area, without involvement of the pulmonary parenchyma. This behaviour suggests a collagenolytic activity, similar to the Actinomyces israelii.

Our patient didn’t have a periodontal disease but presented dental caries, in our search 12/20 patients had periodontal disease or dental caries, in one patient was not mentioned.

The diagnosis of Aa depends on bacterial culture, the major number of cases have been diagnosed by culture of biopsy specimen or aspirate from the involved tissue, isolation and identification of this organism may require prolonged incubation (72– 96 h) [24]. The use of modern molecular diagnostic methods may be more rapid and accurate.

Aa is generally susceptible to main classes of antibiotics (cephalosporins, aminoglycosides, chloramphenicol, rifampicin and tetracycline). Resistance to ampicillin and penicillin is common. In addition to antimicrobial therapy, dental medication will be needed to avoid recurrence.

The optimal duration of therapy is not known, but a long period of antibiotic therapy is needed. Duration depends on the clinical response of the patient, on the extent of tissue involvement, and on resolution of the infective process on follow-up.

In the pediatric cases reviewed here, the range of duration of treatment was between 21 to 365 days in survived children, in our patient it lasted 180 days. The major number of children survived (18/20), without sequelae. Two patients died as a result of a compromise of the general conditions induced by the underlying disease (cerebral palsy) [8, 14].

In conclusion extra-oral infections caused by Aa are extremely rare and not yet well described in current literature, especially in pediatric patients. Our patient represents the only pediatric case described in literature presenting with subphrenic abscess and pulmonary empyema without involvement of lung parenchyma. Aa pulmonary involvement is quite rare in children and can mimic tuberculosis or malignancy. Patients are usually immuno-competent and most of them have an underlying periodontal disease or dental caries. Diagnosis is not prompt and requires tissue biopsy and prolonged bacterial culture. Duration of antibiotic treatment is usually prolonged and based on clinical and radiological response. Our case report reminds us the importance of an accurate inspection of the oral cavity during the examination of pediatric patients and of considering HACEK group infections among differential diagnosis in children with suggestive clinical signs.

## Data Availability

Not applicable.
